# Interlimb and Intralimb Synergy Modeling for Lower Limb Assistive Devices: Modeling Methods and Feature Selection

**DOI:** 10.34133/cbsystems.0122

**Published:** 2024-07-03

**Authors:** Fengyan Liang, Lifen Mo, Yiou Sun, Cheng Guo, Fei Gao, Wei-Hsin Liao, Junyi Cao, Binbin Li, Zhenhua Song, Dong Wang, Ming Yin

**Affiliations:** ^1^ State Key Laboratory of Digital Medical Engineering, School of Biomedical Engineering, Hainan University, Sanya, China.; ^2^ Key Laboratory of Biomedical Engineering of Hainan Province, One Health Institute, Hainan University, Sanya, China.; ^3^ Department of Rehabilitation Medicine, Affiliated Haikou Hospital of Xiangya Medical College, Central South University, Haikou, China.; ^4^ Shenzhen Institute of Advanced Technology, Chinese Academy of Sciences, Shenzhen, China.; ^5^ Department of Mechanical and Automation Engineering, The Chinese University of Hong Kong, Shatin, China.; ^6^ Key Laboratory of Education Ministry for Modern Design and Rotor-Bearing System, School of Mechanical Engineering, Xi’an Jiaotong University,Xi’an, China.

## Abstract

The concept of gait synergy provides novel human–machine interfaces and has been applied to the control of lower limb assistive devices, such as powered prostheses and exoskeletons. Specifically, on the basis of gait synergy, the assistive device can generate/predict the appropriate reference trajectories precisely for the affected or missing parts from the motions of sound parts of the patients. Optimal modeling for gait synergy methods that involves optimal combinations of features (inputs) is required to achieve synergic trajectories that improve human–machine interaction. However, previous studies lack thorough discussions on the optimal methods for synergy modeling. In addition, feature selection (FS) that is crucial for reducing data dimensionality and improving modeling quality has often been neglected in previous studies. Here, we comprehensively investigated modeling methods and FS using 4 up-to-date neural networks: sequence-to-sequence (Seq2Seq), long short-term memory (LSTM), recurrent neural network (RNN), and gated recurrent unit (GRU). We also conducted complete FS using 3 commonly used methods: random forest, information gain, and Pearson correlation. Our findings reveal that Seq2Seq (mean absolute error: 0.404° and 0.596°, respectively) outperforms LSTM, RNN, and GRU for both interlimb and intralimb synergy modeling. Furthermore, FS is proven to significantly improve Seq2Seq’s modeling performance (*P* < 0.05). FS-Seq2Seq even outperforms methods used in existing studies. Therefore, we propose FS-Seq2Seq as a 2-stage strategy for gait synergy modeling in lower limb assistive devices with the aim of achieving synergic and user-adaptive trajectories that improve human–machine interactions.

## Introduction

**Fig. 1.**
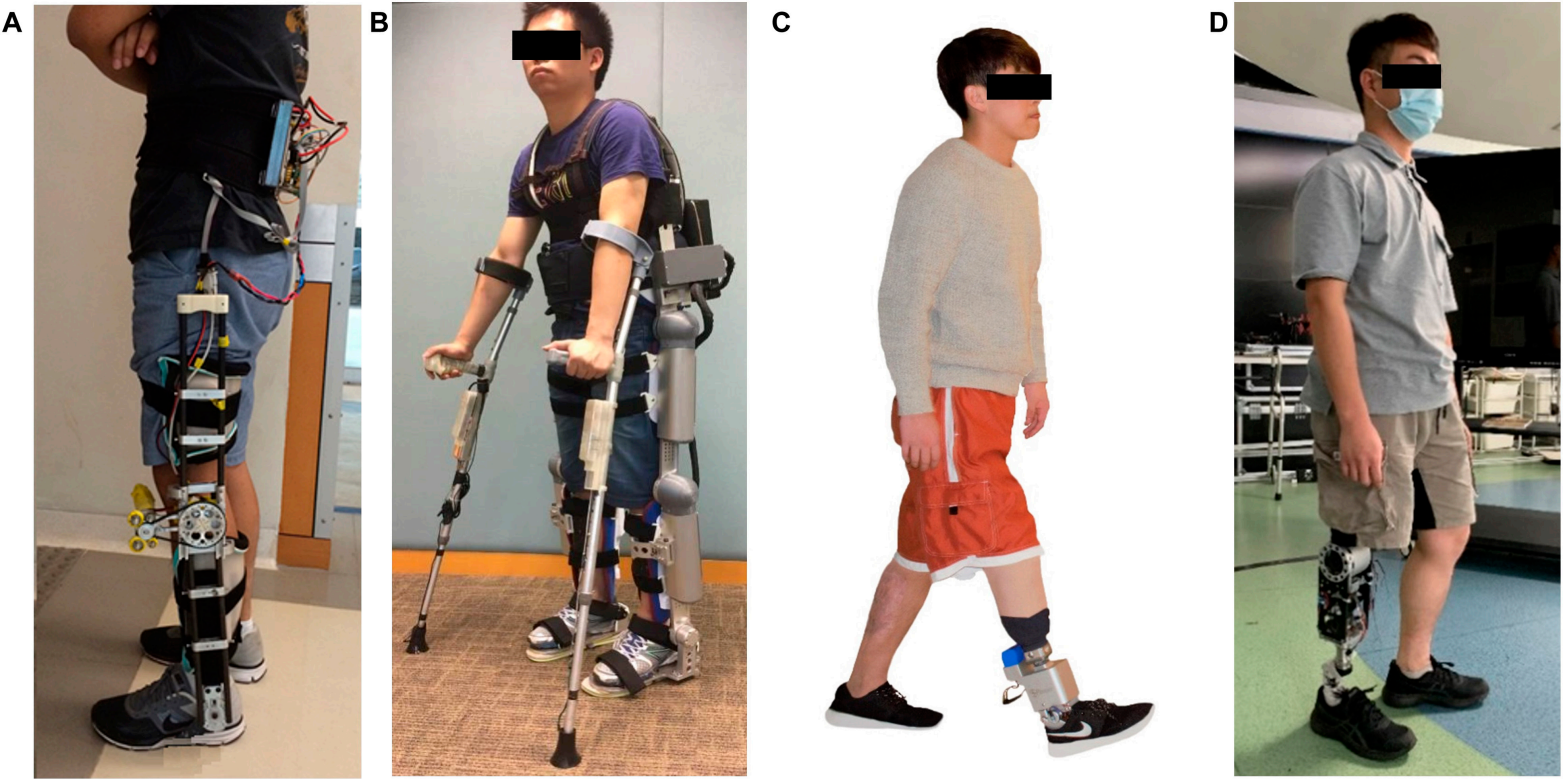
Our self-developed assistive devices. (A) Assistive knee brace [11], (B) CUHK-EXO [10], (C) ankle–foot prosthesis [12], and (D) transfemoral prosthesis [13,14].

Various lower limb assistive devices have been designed and developed with the aim of restoring or enhancing motor function in patients with mobility problems [1,2]. Over the past few decades, a range of lower limb assistive devices have been developed, including full lower limb exoskeletons (e.g., HAL [3], ReWalk [4], Ekso GT [5], and LOKOMAT [6]), partial lower limb exoskeletons or powered orthoses [e.g., C-Brace system (Ottobock, Duderstadt, Germany) and Exoskeleton Ankle Robot] [7], powered ankle–foot prostheses [8], and powered transfemoral prostheses [9]. Our research group has also developed several lower limb assistive devices, including a lower limb exoskeleton for individuals with paralysis, an assistive knee brace for those experiencing knee problems, a powered ankle–foot prosthesis for transtibial amputees, and a powered prosthetic knee for transfemoral amputees (Fig. 1) [10–14]. Patients with limited or disabled mobilities, including amputees, stroke survivors, and individuals with spinal cord injuries, have various medical conditions. Designing the reference trajectories of assistive devices based on the gait of healthy individuals necessitates tuning, adjusting, and limiting the natural variability of gait [1,15], which cannot meet the needs of different patients with different medical conditions. Therefore, control of active assistive devices is a critical and challenging issue when designing and generating user-, temporal-, and phase-adaptive and synergistic reference trajectories for various patients.

During walking, we normally move our arms and legs concurrently in a coordinated manner. From a neuroscience perspective, synergies or interjoint coordination and cooperation form different human motions, including locomotion. The central nervous system combines and coordinates different synergies to perform different tasks [2]. More specifically, the descending supraspinal signals are known to modulate the coordination and timing of muscle activities in the 4 limbs concurrently during mammalian locomotion [16]. These concepts are believed to have implications for locomotor rehabilitation [17].

Joint synergy, which is the modular coordination of lower limb joints during walking, has been explored since the 1990s; it includes both interlimb (joint coordination between 2 limbs) and intralimb (joint coordination within one limb) synergies. Borghese et al. [18] found planar covariations between the hip, knee, and ankle joints when walking at different speeds, suggesting the central nervous system’s role in maintaining kinematic invariance. Principal components analysis (PCA) has emerged as the most frequently used mathematical approach for building synergy models [19]. For example, Bockemühl et al. [20] demonstrated that the first 3 principal components often account for more than 97% of the variance in synergy models. However, there have been concerns about PCA’s limited modeling performance when applied to synergies (for example, the mean distortion rate in knee joint estimation is 29.13%) [15,21–24], which may hinder its practical applications.

The concept of synergy empowers novel human–machine interfaces and has been applied to predict the trajectories of lower limb assistive devices (Table 1). The aim here is to anticipate appropriate reference trajectories for the affected or missing body parts from the motions of the intact parts using a specific relationship, either interlimb or intralimb synergy. This relationship is modeled from the kinematics of able-bodied individuals, enabling utilization of their synergies to guide patients toward rehabilitation and successful walking. For hemiplegic patients, the sound parts refer to the legs on their sound side, whereas for amputees, the sound parts refer to their residual limbs. During assisted walking, positive feedback can be sent to the patients’ nervous systems to facilitate brain recovery. This approach shows promise in enhancing patient outcomes and presents a valuable contribution to the field of assistive technologies [21–23].

**Table 1.** Differences between interlimb and intralimb synergies and their applications

	Interlimb synergy	Intralimb synergy
Meaning	Joint coordination relationships among joints in the two lower limbs during locomotion	Joint coordination relationships among joints within one of the lower limbs during locomotion
Application	Exoskeleton	Orthosis, prosthesis
Device user	Stroke patient (most of whom are hemiplegic)	People with knee problems and above-knee amputees
Impaired part	One of the limbs	A part of one limb
Goal	Generate synergic reference trajectories from the motion of sound side to provide adaptive guidance to stroke patients, encourage participation and help with rehabilitation	Generate synergic reference trajectories in line with the wearer’s movements for autonomous control

On the one hand, trajectory prediction based on interlimb synergy has been proposed for rehabilitative exoskeletons: The traditional trajectory generation method for these rehabilitative exoskeletons involves using the gait data of able-bodied individuals and tuning, neglecting the variabilities under patients with stroke and different situations. In synergy-based trajectory prediction, the reference trajectory of the affected side is produced online from the kinematics of the sound side based on interlimb synergy. Thus, the generated trajectory is user-adaptive and in line with the user’s motion. This approach is called complementary limb motion estimation [21] and has undergone validation in clinical trials, with demonstrated benefits such as reduced interference on the wearer, energy savings for exoskeletons, and increased patient participation [21,24]; here, patient participation is a crucial aspect of rehabilitation.

However, there is still room for improvement in the estimation performance of interlimb synergy modeling, considering its poor trajectory prediction, particularly for the knee joint [21]. Previous studies have explored various methods, such as PCA, best linear unbiased estimation (BLUE), and long short-term memory (LSTM), to improve the estimation performance during synergy modeling [22–24]. In our previous work [15], we introduced LSTM for the first time to model the interlimb synergy and generate reference trajectories for our self-designed exoskeleton CUHK-EXO.

On the other hand, trajectory prediction based on intralimb synergy has also been proposed for partial limb exoskeletons and above-knee prostheses. The traditional approach for these devices is the finite state impedance control, where state machines are used to determine the gait percent, walking speed, and switching rules, and the wearers have to adapt to the devices. However, in synergy-based trajectory generation, the trajectory of the active prosthesis is predicted online from the wearer’s residual limb. Note that the wearer still maintains control over the residual limb, so this approach offers direct device control and instant responses to motion intent. Various methods have been investigated to estimate knee and hip angles based on shank/thigh motion data to obtain a user-dominated gait. Our previous work [1] proposed the use of thigh kinematics to estimate knee angles based on intralimb coordination, using an LSTM to model intralimb synergy. A functional method for controlling active knee prostheses called synergy-based knee angle estimation based on thigh motion using Gaussian process regression, was introduced by Eslamy and Schilling [25]. In their intersubject tests, they achieved an average root mean square error (RMSE) of 6.36°, mean absolute error (MAE) of 5.28°, and *R*^2^ of 0.89. However, further research is needed to enhance the synergy modeling and estimation performance of this method.

To improve modeling performances and obtain more synergic synergy-based trajectories, an optimal synergy modeling method is required for both interlimb and intralimb synergies. PCA was first suggested as a synergy modeling method in 2004 [26] and has since become the most commonly used approach [19]. Over the years, various statistical and neural network methods have been used to model intralimb and interlimb synergies, including PCA [21], BLUE [22], LSTM [15], and feed forward neural network (FFNN) [27], as detailed in Table 2. Among these methods, LSTM has demonstrated superior performance compared to PCA in both intralimb and interlimb synergy modeling [1,2]. Although Zou et al. [28] suggested that sequence-to-sequence (Seq2Seq) may be more effective than LSTM for interlimb synergy modeling, and the diverse scales and datasets used in various studies make it challenging to quantify and compare the results as these studies have all used different datasets. Therefore, no comprehensive comparisons have been conducted, and no optimal conclusions have been obtained from existing studies regarding the best synergy modeling method.

**Table 2.** Synergy modeling methods in extant studies

	Synergy	Sensor	Input	Method	FS
Vallery 2006 [21]	Interlimb	Vicon mocap system	Hip and knee angle of the ipsilateral leg	PCA	/
Vallery 2008 [22]	Interlimb	Potentiometers	Hip and knee angle of the ipsilateral leg	BLUE	/
Vallery 2011 [23]	Interlimb	Goniometer and gyroscopes	Hip and knee angle and angular velocities of the ipsilateral leg	BLUE	/
Hassan 2014 [24]	Interlimb	IMUs	Joint angles and angular velocities of upper and lower limbs	PCA	/
Chereshnev 2018 [58]	Interlimb	Accelerometers and gyroscopes	Thigh angles and angular velocities of 2 legs	RNN	/
Liang 2018 [15]	Interlimb	IMUs	Joint angles and angular velocities of the ipsilateral leg	LSTM	/
Liu 2016 [60]	Interlimb and intralimb	Encoder	Hip and ankle angles of 2 legs	LSTM	/
Lim 2019 [27]	Interlimb and intralimb	IMU	Accelerations, velocities, and displacements at sacrum	FFNN	/
Bennett 2013 [36]	Intralimb	IMUs	Shank and thigh accelerations and angular velocities of the ipsilateral leg	ANN	Not thorough
Zhang 2010 [62]	Intralimb	Accelerometers	Thigh accelerations	Gaussian particle filter	/
Eslamy 2018 [25]	Intralimb	Qualisys mocap system	Shank angle and angular velocities of the ipsilateral leg	Gaussian process regression	Not thorough
Eslamy 2020 [37]	Intralimb	IMUs	Thigh angles and angular velocities of the ipsilateral leg	Gaussian process regression	Not thorough
Zou 2021 [28]	Interlimb	IMUs	Hip & knee angle of the ipsilateral leg	Seq2Seq	/
Liang 2021 [1]	Intralimb	IMU	Thigh accelerations and angular velocities of the ipsilateral leg	LSTM	Not thorough
Quintero 2018 [61]	Intralimb	IMU	Thigh angular position	Discrete Fourier transform	/
Rai 2019 [59]	Interlimb and intralimb	IMUs	Hip and ankle angles of 2 legs	LSTM	/

Seq2Seq models perform well in time-series forecasting and may be optimal for synergy modeling. Recently, Seq2Seq models have shown state-of-the-art performances across various domains [29], including machine translation [30,31], image captioning [32], and motion prediction [33]. The present study aims to comprehensively compare modeling methods, including Seq2Seq, LSTM, recurrent neural network (RNN), and gated recurrent unit (GRU) neural network using the same dataset to determine the optimal method for both interlimb and intralimb synergy modeling. This study aims to contribute to the current understanding of synergy modeling and its practical application in obtaining synergic (user-adaptive) and effective trajectories for assistive devices, such as exoskeletons and prostheses.

Feature selection (FS) is another consideration in synergy modeling. In real-world learning problems, data modeling normally involves numerous features, where only a few may correlate with the target. Thus, FS (finding the suitable variables that exhibit good specificity and sensitivity for modeling the target) can help reduce data dimensionality, speed up the learning procedure, and improve modeling quality [34,35]. Synergy modeling is a multivariable regression problem that exploits the correlations between joint angles and various kinematics in the contralateral or ipsilateral leg. Table 2 summarizes 4 studies [1,25,36,37] in which subsets of variables were selected to build different models. However, their FS processes were often limited and incomplete. To solve this limitation, we propose a comprehensive FS process using techniques such as random forest, information gain, and Pearson correlation, aiming to identify and select the most relevant features or those that make the most contributions to the output (i.e., joint angle). This approach ultimately improves modeling performance, reduces model complexity and minimizes computational load and time, thereby allowing more feasible real-world applications.

The results of this study show that Seq2Seq outperforms LSTM, RNN, and GRU in both interlimb and intralimb synergy modeling. Further, FS significantly improves Seq2Seq’s modeling performance. The FS-Seq2Seq (feature selection–based Seq2Seq) yields the best results of synergy modeling. Consequently, a 2-stage strategy, FS-Seq2Seq, is proposed for gait synergy modeling in trajectory generation on assistive devices. Previous synergy modeling studies have not comprehensively compared the modeling methods and neglected the FS processes. This study emphasizes the promise of synergy-based trajectory prediction for assistive devices and provides insights into achieving optimal modeling with optimal feature combinations, resulting in synergic and user-adaptive trajectories that improve human–machine interactions.

## Materials and Methods

The general idea of synergy-based trajectory prediction or generation for lower limb assistive devices involves building a synergy model from the kinematics of a cohort of able-bodied subjects. This model is then used to generate suitable reference trajectories for the affected parts of the patients using the device based on synergy using data from the sound parts of the patients. Here, we aim to design a synergy-based trajectory generation method for our self-developed assistive devices (Fig. 1), where the first step is to design experiments on gait to obtain adequate training data.

### Gait data acquisition

In the designed gait experiments, a group of 16 healthy male subjects of various heights, weights, ages (mean height, 1.73 ± 0.05 m; mean weight, 64.2 ± 6.7 kg), and no gait-related issues were recruited to walk and return on level ground (a 12-m walkway). Prior to the experiments, written informed consent was obtained from each subject, and the study received ethical approval from the institutional review board of Affiliated Haikou Hospital of Xiangya Medical College, Central South University (register number: SC2022-0088). During the experiments, the subjects were allowed to walk at their own comfortable speeds without speech interference. Each subject performed 5 trials with sufficient rest between the trials.

**Fig. 2.**
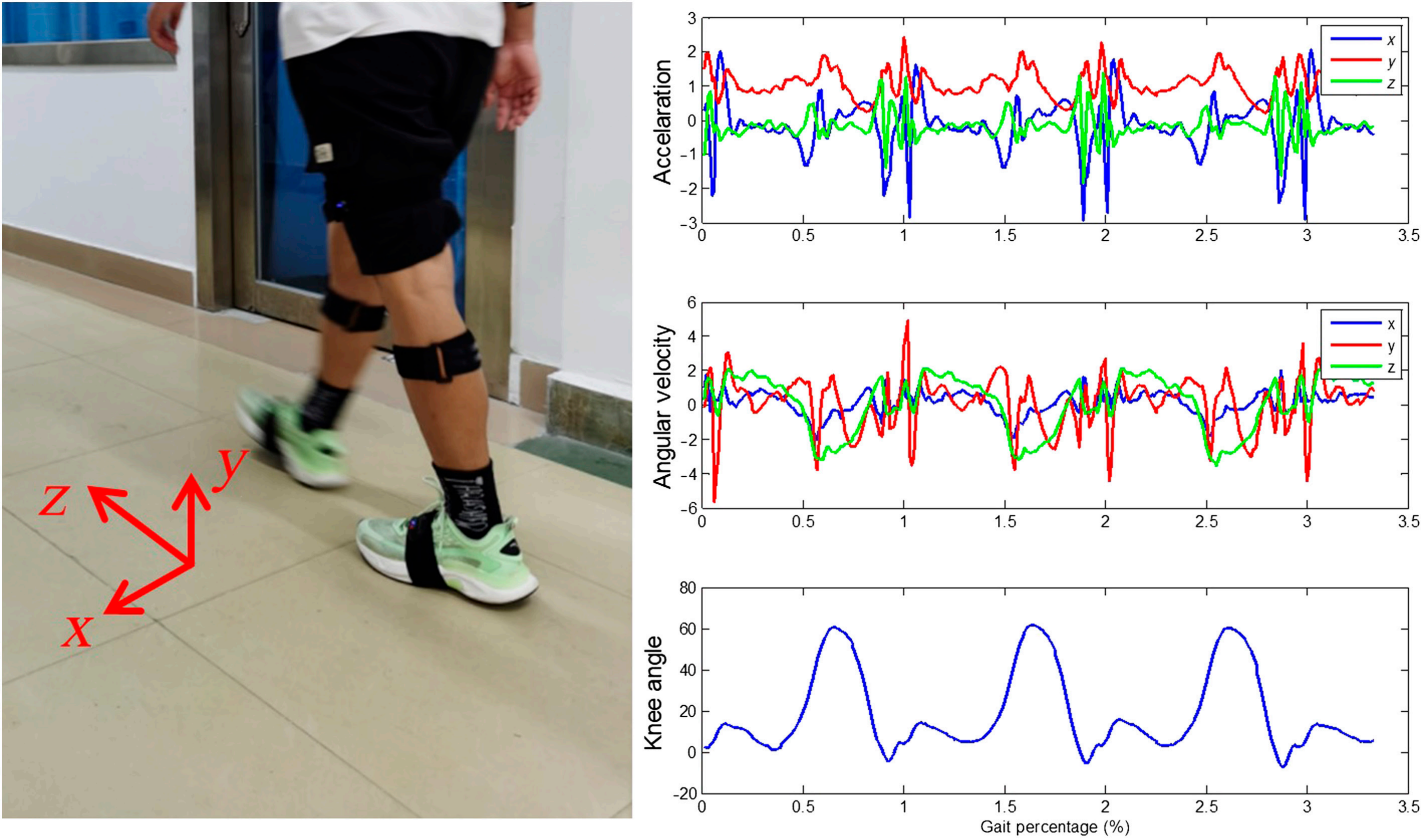
The wearable motion capture system and measured acceleration, angular velocity, and knee angle.

To capture the subjects’ kinematics during walking, a wearable motion capture system (Perception Neuron 3.0 Pro, Noitom, Beijing, China) was used. This system comprising inertial measurement units (IMUs) can capture various parameters, such as accelerations, velocity, angular velocity, position, quaternion, and joint angle. The lower-body module of the wearable system includes 7 IMUs: one at the back of the hip above the coccyx (L3) and 6 distributed over the middle of the shank, thigh, and foot of both legs, as shown in Fig. 2. To secure the IMU sensors, tight straps with velcros were used. It should be emphasized that only the IMUs over the thighs were parallel to the sagittal plane, whereas the other IMUs were positioned forward as per the manual. The output followed the BioVision Hierarchical format, a standard format for motion capture data. The coordinate system was defined as right-handed, as shown in Fig. 2. The forward direction of the subject is defined as the *z* axis, world up direction as the *y* axis, and direction perpendicular to both the *y* axis and *z* axis as the *x* axis. The system recorded data at a sampling frequency of 60 Hz, and calibrations were performed before each trial as per the procedures outlined in the manual. This ensured accurate and reliable measurements of the subjects’ kinematics during the gait experiments.

During the gait experiments, the kinematics of the complete gait cycles of all trials of the 16 subjects were recorded, excluding the first and last 2 steps. A gait cycle is initiated with the heel strike of the right foot and ends with the subsequent heel strike of the right foot. In total, around 1,300 gait cycles were recorded from the 16 subjects, forming a substantial dataset for further analyses and modeling. Before proceeding with the synergy modeling, a standardization step was conducted on the obtained data based on the mean (*σ*) and SD (*μ*) as follows:

$$\;z_{i}=\frac{x_{i}-\mu }{\sigma }$$ (1)

### Feature selection

Various methods have been proposed for FS, including the random forest algorithm [38,39], Pearson correlation (or heatmap) [40,41], and information gain [42,43]. It is worth noting that there is no universally optimal FS method that may be applied to all datasets or problems [44]. The choice of method depends on the specific dataset and modeling objectives. Here, we used 3 commonly used methods, namely, random forest, Pearson correlation, and information gain to conduct systematic experiments to identify the most suitable features or inputs for synergy modeling. The advisable features and the optimal number of features may vary depending on the specific modeling method chosen. Therefore, it is necessary to explore and experiment with different FS methods to determine the most appropriate features for a given modeling scenario.

#### Random forest algorithm

Random forest is one of the important methods used for FS [45,46]. The formulas (Eqs. 2 to 6) describe the calculations in the random forest algorithm for FS:

$${\mathrm{GI}}_{m}=\sum_{k=1}^{\left|K\right|}\sum_{k^{\prime }\neq k}p_{\mathrm{mk}}p_{mk^{\prime }}=1-\sum_{k=1}^{\left|K\right|}p_{\mathrm{mk}}^{2}$$ (2)

$${\mathrm{VIM}}_{\mathrm{jm}}^{\left(\text{Gini}\right)}={\mathrm{GI}}_{m}-{\mathrm{GI}}_{l}-{\mathrm{GI}}_{r}$$ (3)

$${\mathrm{VIM}}_{\mathrm{ij}}^{\left(\text{Gini}\right)}=\sum_{m\in M}\mathrm{VI}M_{\mathrm{jm}}^{\left(\text{Gini}\right)}$$ (4)

$${\mathrm{VIM}}_{j}^{\left(\text{Gini}\right)}=\sum_{i=1}^{n}{\mathrm{VIM}}_{\mathrm{ij}}^{\left(\text{Gini}\right)}$$ (5)

$${\mathrm{VIM}}_{j}=\frac{{\mathrm{VIM}}_{j}}{\sum_{i=1}^{c}{\mathrm{VIM}}_{i}}$$ (6)

where *GI_m_* represents the Gini index of node *m*, *K* denotes the number of classes, and *p_mk_* is the weight of *k* within node *m*. The variable importance measures (VIMs) represent the scores indicating the importance of each of features; specifically, the importance of feature *X_j_* in the *i*th tree is denoted as ${\text{VIM}}_{ij}^{\left(\text{Gini}\right)}$. Assuming that there are *n* trees in the random forest, the importance of *X_j_* is ${\text{VIM}}_{ij}^{\left(\text{Gini}\right)}$. In addition, by normalizing all the obtained importance scores, *VIM_j_* is derived. Consequently, the Gini importance, *VIM_j_*, can effectively quantify the importance of each feature in the random forest.

#### Pearson correlation

The Pearson correlation (PC) is a statistical measure of linear correlation between 2 sets of data, where a value close to 1 or −1 indicates a stronger correlation. FS can be performed by evaluating the PCs between the input features and a target variable. Given 2 variables *X*_1_ and *X*_2_, the PC is calculated is as follows:

$$\begin{array}{c} \rho_{x_{1}x_{2}}=\\ \frac{\mathrm{Cov}\left(X_{1},X_{2}\right)}{\sigma_{X_{1}}\sigma_{X_{2}}}=\frac{{\mathrm{EX}}_{1}X_{2}-{\mathrm{EX}}_{1}*X_{2}}{\sqrt{E\left(X_{1}^{2}\right)-E^{2}\left(X_{1}\right)}\sqrt{E\left(X_{2}^{2}\right)-E^{2}\left(X_{2}\right)}} \end{array}$$ (7)

#### Information gain

The information gain measures the amount of information that each feature provides regarding the outcome [47,48] and is defined as

$$\text{Gains}\left(U,V\right)=\mathrm{Ent}\left(U\right)-\mathrm{Ent}\left(U|V\right)$$ (8)

where the prior entropy *Ent*(*U*) represents the level of uncertainty before transmitting information *U*, whereas the posterior entropy *Ent*(*U*| *V*) represents the average uncertainty remaining after receiving information *V*. During the FS process, the target variable (output) is considered as information *U*, and the feature variable is information *V*. Different information gains are calculated by incorporating different information *V*. A higher information gain indicates that the variable has a stronger ability to reduce uncertainty. Thus, features can be sorted and selected on the basis of their respective information gains.

### Synergy modeling

In the modeling experiments, 4 different neural networks were used: LSTM, Seq2Seq, RNN, and GRU. These were used to model both interlimb and intralimb synergies based on the kinematic data. Here, interlimb synergy refers to the mapping from the kinematics of the right lower limb (including accelerations, velocities, angular velocities, positions of the right shank and thigh, and the right hip and knee angles) to the hip angles of the left side; this means that using the information from the right side, the model can predict the corresponding hip angles of the left side. Correspondingly, intralimb synergy is defined as the mapping from the accelerations, velocities, angular velocities, and positions of the left thigh to left knee angles; in other words, the model uses the information of the left thigh to predict the ipsilateral knee angles.

To assess the universality of the synergy models, the gait data of all 16 subjects were utilized for intersubject experiments. The leave-one-out cross-validation method was used in this process, which involves selecting one subject’s gait data for testing and using the remaining 15 subjects’ data for training. For each testing iteration, the joint angles of the selected subject were estimated on the basis of the synergy models derived from the training data. The resulting hip trajectory was then compared with the actual measured trajectory. This process was repeated 16 times by utilizing each subject’s data as the test data once.

The number of epochs in each neural network training session was set to 10 to guarantee fair comparison. Furthermore, 4 different scales (RMSE, Pearson correlation, *R*^2^, and MAE) were used to quantify and compare the model performances. The mean results were calculated by averaging the values of the 4 metrics obtained from the 16 experimental runs.

#### Seq2Seq

Seq2Seq is an encoder–decoder network designed for handling sequences with variable-length inputs and outputs [49]. Seq2Seq is usually combined with LSTM; however, the performance of Seq2Seq degrades as the input sequence length increases. To address this limitation, an attention mechanism was incorporated in the network. In a previous study by Zou et al. [28], Seq2Seq was used with an attention mechanism to model interlimb synergy, where LSTM served as the basic neural unit for both the encoder and decoder. Qin et al. [50] introduced the dual-stage attention-based RNN, which demonstrated improved efficacy as a Seq2Seq encoder–decoder network by incorporating attention mechanisms into both the encoder and decoder stages. In the present study, we aim to enhance synergy modeling using dual-stage attention-based RNN, with the encoder and decoder models based on LSTM (similar to 2.2.2). Equations 9 to 16 represent the algorithm for the attention mechanism:

$$e_{t}^{k}=v_{e}^{T}\text{tanh}\left(W_{e}\left[h_{t-1};s_{t-1}\right]+U_{e}x_{k}\right.$$ (9)

$$\alpha_{t}^{k}=\frac{\text{exp}\left(e_{t}^{k}\right)}{\sum_{i=1}^{n}\text{exp}\left(e_{t}^{i}\right)}$$ (10)

$${x\prime }_{t}={\left(\alpha_{t}^{1}x_{t}^{1},\alpha_{t}^{2}x_{t}^{2},\cdots ,\alpha_{t}^{n}x_{t}^{n}\right)}^{T}$$ (11)

$$h_{t}=f_{1}\left(h_{t-1},{x\prime }_{t}\right)$$ (12)

$$\beta_{t}^{i}=v_{d}^{T}\text{tanh}\left(W_{d}\left[d_{t-1};{s\prime }_{t-1}\right]+U_{d}h_{i}\right),1\leq i\leq T$$ (13)

$$w_{t}^{i}=\frac{\text{exp}\left(\beta_{t}^{i}\right)}{\sum_{j=1}^{T}\text{exp}\left(\beta_{t}^{j}\right)}$$ (14)

$$\;c_{t}=\sum_{i=1}^{T}w_{t}^{i}h_{i}$$ (15)

$${y\prime }_{t-1}{w\prime }^{T}\left[y_{t-1};c_{t-1}\right]+b\prime$$ (16)

where *v_e_*, *W_e_*, and *U_e_* denote the parameters to be learned and $\alpha_{t}^{k}$ is the attention weight; Eqs. 9 to 11 establish the attention mechanism of the encoder, and Eqs. 13 to 16 represent the attention mechanism of the decoder. During the encoding process, the input is denoted as *x*, and the output is *x′*. Unlike LSTM, the hidden state of the encoder *h_t_* is obtained by applying *x′* and *h*_*t*−1_ as inputs to the LSTM unit *f*_1_ in Eq. 12. The attention weight of the encoder’s hidden state is then updated on the basis of the decoder’s hidden state *d*_*t*−1_ and LSTM state ${s\prime }_{t-1}$ using Eq. 13. In Eqs. 13 to 16, *V_d_*, *W_d_*, and *U_d_* are parameters to be learned. Finally, the combined target series ${y\prime }_{t-1}$ is obtained as shown in Eq. 16.

**Fig. 3.**
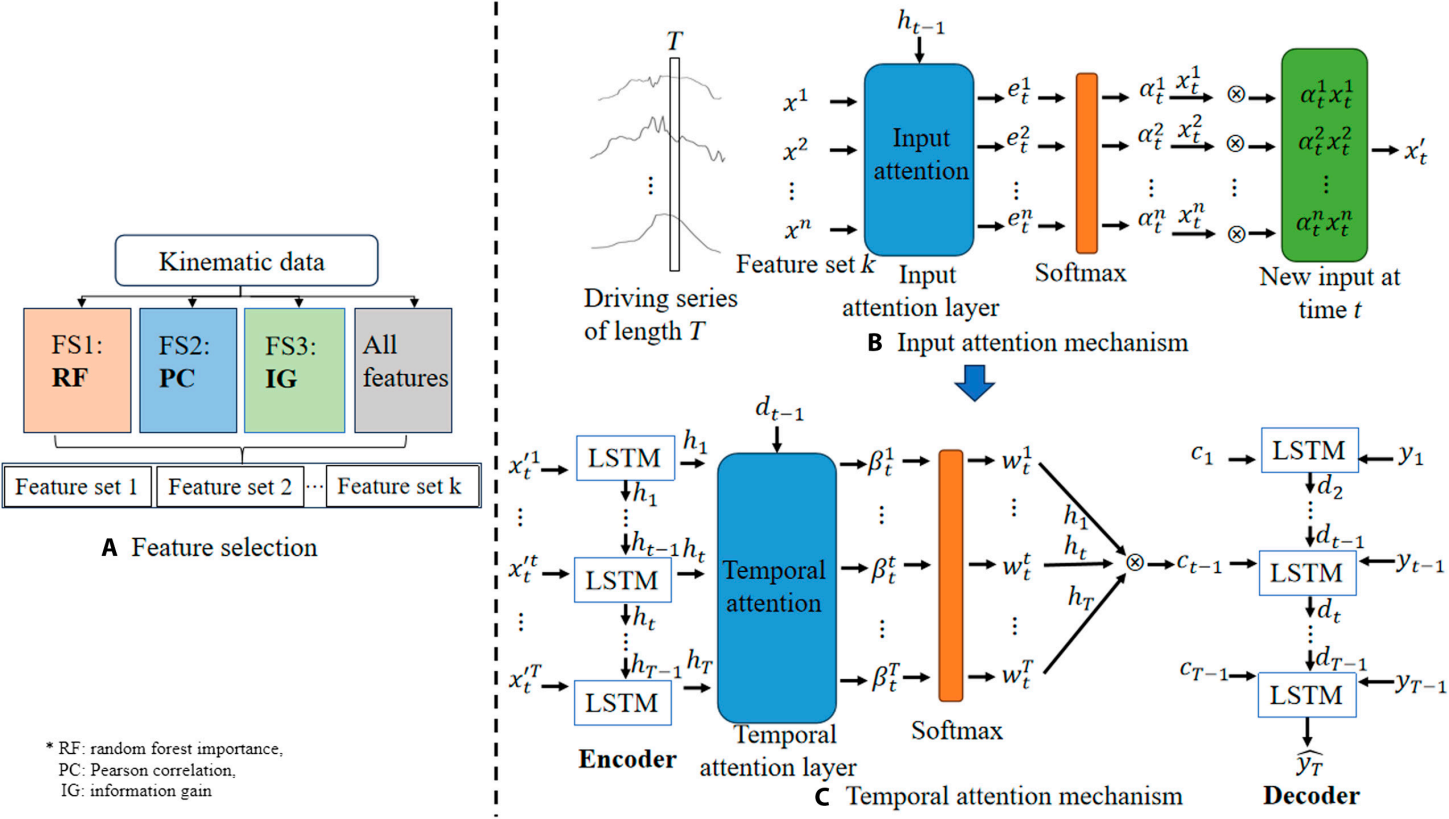
FS-Seq2Seq flow chart. (A) Feature selection. (B) Input attention mechanism. (C) Temporal attention mechanism.

Figure 3 shows the flowchart of FS-Seq2Seq, as a 2-stage strategy. In the FS stage, 3 FS methods are used to choose the input motion data. The second stage involves a Seq2Seq model, which comprises 2 phases. During the first phase, the attention mechanism is utilized to adaptively extract relevant features at each moment, serving as the input for the encoder. During the second phase, another attention mechanism is used to select the related encoder hidden states. The FS-Seq2Seq model requires the determination of 3 parameters: window size *T*, size of the hidden states *P* for the encoder, and size of the hidden states *Q* for the decoder. The window size *T* is set to 20. Usually, the sizes of the hidden units for the encoder and decoder are set the same for simplicity; here, we set *P* = *Q* = 128. Regarding the training parameters, we specify the number of training epochs as 10, and the learning rate is set to 0.001. It is worth noting that the parameters set for models with or without FS, such as FS-Seq2Seq and Seq2Seq, are the same. For the RNN, LSTM, and GRU models, it is necessary to specify the sizes of the hidden units *E*. Similar to the encoder/decoder in FS-Seq2Seq, we set *E* = *P* = *Q* = 128 to ensure the same complexity of the hidden layer. In addition, to ensure fair comparison conditions, the number of epochs and learning rates of these 3 models are set identical to those of the FS-Seq2Seq model.

#### RNN

RNNs are a type of neural network commonly used in natural language processing. They have hidden layers that allow earlier outputs to be used as inputs, enabling the modeling of sequential data effectively. Traditional RNNs have a simple internal structure and low computational requirements. The RNN is defined in Eqs. 17 and 18:

$$o_{t}=g\left(Vs_{t}+b_{2}\right)$$ (17)

$$s_{t}=f\left(Ux_{t}+Ws_{t-1}+b_{1}\right)$$ (18)

#### LSTM

LSTM was first introduced in 1997 [51], as a modified version of RNN that exceled at learning and retaining past information. It has now become a popular tool for various tasks involving time-series information, such as classifying, processing, and estimating [52,53]. An LSTM unit normally includes 3 gates: input, output, and forget gates. These gates control the flow of information within the LSTM unit. Equations 19 to 23 illustrate the basic equations of the LSTM.

$$f^{\left(t\right)}=\sigma \left(W_{1}^{f}x^{\left(t\right)}+W_{2}^{f}h^{\left(t-1\right)}b_{f}\right)$$ (19)

$$s^{\left(t\right)}=g^{\left(t\right)}⊙i^{\left(t\right)}+s^{\left(t-1\right)}⊙f^{\left(t\right)}$$ (20)

$$i^{\left(t\right)}=\sigma \left(W_{1}^{i}x^{\left(t\right)}+W_{2}^{i}h^{\left(t-1\right)}b_{i}\right)$$ (21)

$$o^{\left(t\right)}=\sigma \left(W_{1}^{o}x^{\left(t\right)}+W_{2}^{o}h^{\left(t-1\right)}b_{o}\right)$$ (22)

$$h^{\left(t\right)}=\text{tanh}\left(s^{\left(t\right)}\right)⊙o^{\left(t\right)}$$ (23)

The input and output gates denoted by *i*^(*t*)^ and *o*^(*t*)^, respectively, determine which values are to be stored in or outputted from the memory. The forget gate *f*^(*t*)^ decides the values that be removed from the memory block. The sigmoid function (*σ*) is often used to compute the gating values. After each training session, the connection weights and bias parameters *W*_1_, *W*_2_, and *b* are updated using a process called backpropagation; this process involves calculating the gradients of the loss function with respect to the parameters and using an optimization algorithm (e.g., gradient descent) to update the parameters accordingly.

#### GRU

GRU is another type of RNN applied in diverse areas such as stock prediction [54], gait recognition [55], and gait prediction [56]. Similar to LSTM, GRU was designed to solve the problem of long-term dependencies, but it is computationally simpler than LSTM [57]. The GRU cell is governed by 2 gates: a reset gate *r_t_* and an update gate *z_t_*. Equations 24 to 27 illustrate the algorithm of GRU:

$$r_{t}=\sigma \left(W_{r}\left[h_{t-1};x_{t}\right]\right)$$ (24)

$$z_{t}=\sigma \left(W_{z}\left[h_{t-1};x_{t}\right]\right)$$ (25)

$$h_{t}^{\prime }=\text{tanh}\left(W\left[r_{t}*h_{t-1};x_{t}\right]\right)$$ (26)

$$h_{t}=\left(1-z_{t}\right)*h_{t-1}+z_{t}*h_{t}^{\prime }$$ (27)

where the 2 gate states (*r_t_,z_t_*) are updated on the basis of the input of the current node *x_t_* and hidden state *h*_*t*−1_. Then, the reset gate *r_t_* is used to reset the data, which are concatenated with input *x_t_* and scaled using an activation function tanh to scale the data to the range of (−1, 1). Thus, $h_{t}^{\prime }$ is obtained, and, ultimately, *h_t_* is computed using Eq. 27.

## Results

### FS results

**Fig. 4.**
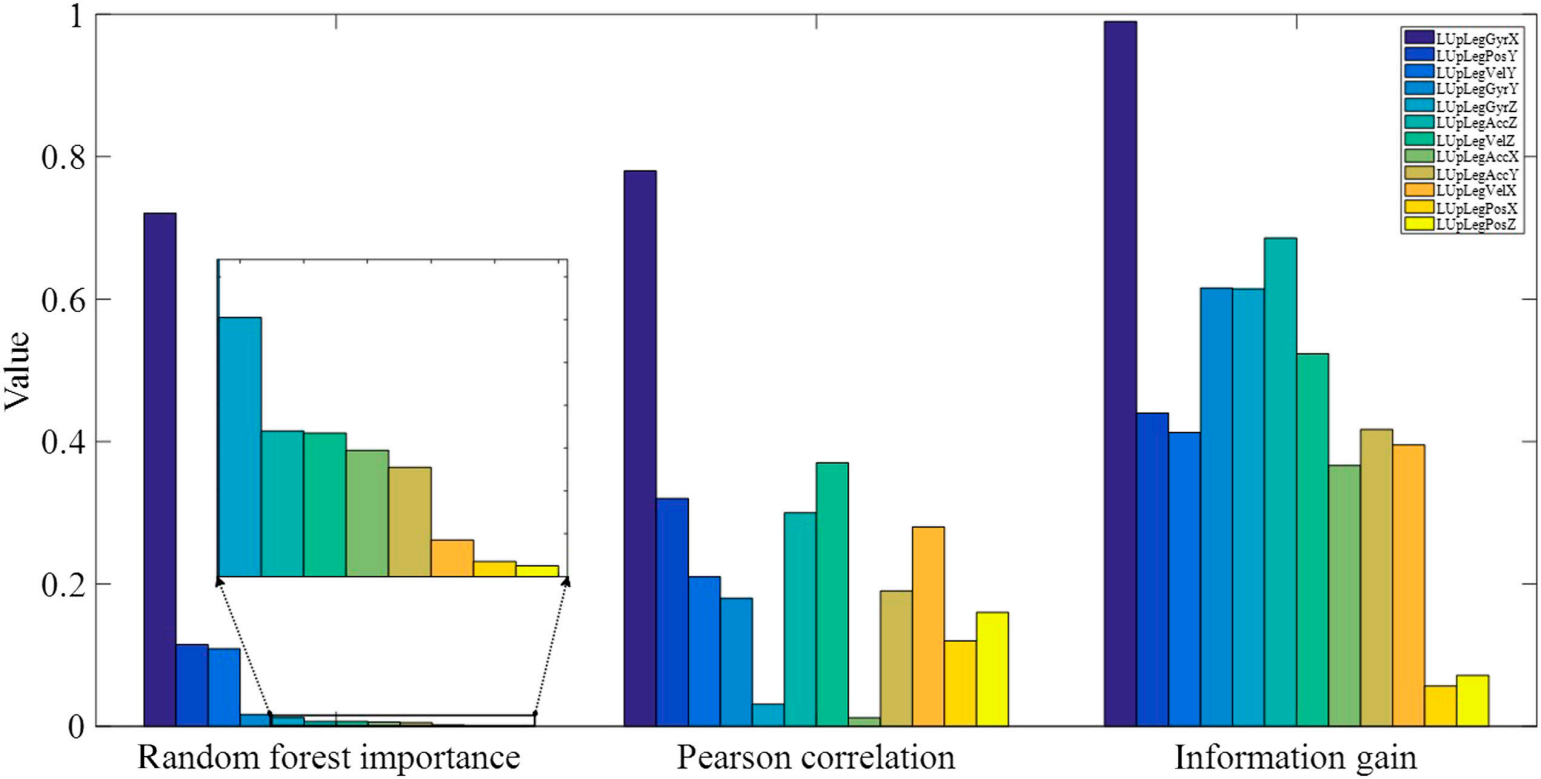
FS results of the random forest, Pearson correlation, and information gain methods for intralimb synergy modeling. *ω_upx_* is top suggested by all 3 methods. The Seq2Seq and RNN models exhibited the lowest error and best fitness values with the top 5 features suggested by the random forest algorithm.

As introduced above, 3 methods were used for FS in both interlimb and intralimb synergy modeling. In intralimb modeling, suitable input features need to be selected from a pool of 12 features [including accelerations (*a*), velocities (*V*), positions (*x*), and angular velocities (*ω*) of the thigh in the 3 directions). To comprehensively analyze and compare the FS results obtained from the random forest algorithm, Pearson correlation (heatmap), and information gain, the results of these 3 methods for intralimb synergy modeling are visualized in one figure (Fig. 4). Only the top 5 features (threshold: random forest importance > 0.01, Pearson correlation > 0.25, and information gain > 0.5) with the highest importance or correlations or information gain are displayed. To maintain the range of 0 to 1, the Pearson correlation values presented here are absolute values.

According to FS results in Fig. 4, the random forest algorithm suggests that *ω_upx_*, the angular velocities in the sagittal plane (perpendicular to the *x* direction) at the right thigh make the largest contributions to the output *θ_LKnee_*. In addition, the Pearson correlation suggests that *ω_upx_* has the strongest correlation with the output. Last, the information gain analysis demonstrates that *ω_upx_* can greatly maximize the information gain. Thus, *ω_upx_* is suggested by all 3 methods. It is worth noting that the random forest and information gain methods select the same top 3 features (*ω_upx_*, *ω_upy_*, and *ω_upz_*). In comparison, *V_upz_* and *a_upz_* are not among the top 5 features selected by random forest but are within the top 5 according to the Pearson correlation and information gain methods.

To determine the optimal combinations of features, experiments were conducted using different combinations of the selected features (*n* = 1 to 5) suggested by the 3 methods (Table 3). The experiments included sessions with one to 5 selected features, as well as a session with all 12 features. Thus, a total of 13 sessions were conducted to explore different feature combinations for intralimb synergy modeling. The results indicate that both Seq2Seq and RNN models performed the best in terms of error and fitness with the input features *ω_upx_ω_upy_ω_upz_V_upy_x_upy_*, which are the top 5 features selected by random forest. However, the LSTM and GRU models using the features obtained by FS are not good for modeling with all 12 features.

**Table 3.** Average experimental results of different combinations of the features selected for intralimb synergy modeling

	Seq2Seq			LSTM			RNN			GRU		
Features	RMSE	MAE	*R* ^2^	RMSE	MAE	*R* ^2^	RMSE	MAE	*R* ^2^	RMSE	MAE	*R* ^2^
*ω_upx_*	0.958	0.684	0.998	1.938	1.445	0.990	2.017	1.544	0.989	1.944	1.440	0.990
*ω_upx_x_upy_*	0.909	0.637	0.998	1.744	1.330	0.992	1.888	1.458	0.991	1.802	1.335	0.992
*ω_upx_v_upz_*	0.918	0.635	0.998	1.874	1.385	0.991	1.896	1.410	0.990	1.892	1.434	0.991
*ω_upx_a_upz_*	0.947	0.666	0.998	1.620	1.204	0.993	1.969	1.474	0.989	1.676	1.236	0.992
*ω_upx_x_upy_v_upy_*	0.900	0.641	0.998	1.625	1.201	0.994	1.828	1.474	0.993	1.584	1.184	0.994
*ω_upx_v_upz_x_upy_*	0.911	0.631	0.998	1.763	1.329	0.992	1.890	1.437	0.991	1.748	1.353	0.992
*ω_upx_a_upz_ω_upy_*	0.913	0.644	0.998	1.571	1.153	0.993	1.942	1.435	0.990	1.667	1.254	0.993
*ω_upx_x_upy_v_upy_ω_upy_*	0.899	0.633	0.998	1.564	1.173	0.994	1.708	1.340	0.993	1.590	1.167	0.994
*ω_upx_v_upz_x_upy_a_upz_*	0.905	0.626	0.998	1.604	1.174	0.994	1.848	1.387	0.991	1.658	1.203	0.993
*ω_upx_a_upz_ω_upy_ω_upz_*	0.922	0.648	0.998	1.539	1.162	0.994	1.793	1.329	0.992	1.593	1.218	0.993
*ω_upx_x_upy_v_upy_ω_upy_ω_upz_*	**0.859**	**0.596**	**0.998**	1.452	1.093	0.995	**1.520**	**1.189**	**0.994**	1.523	1.184	0.995
*ω_upx_v_upz_x_upy_a_upz_v_upx_*	0.901	0.633	0.998	1.570	1.171	0.994	1.918	1.482	0.991	1.575	1.220	0.994
*ω_upx_a_upz_ω_upy_ω_upz_v_upz_*	0.877	0.614	0.998	1.627	1.219	0.994	1.823	1.376	0.992	1.642	1.258	0.993
All	0.902	0.631	0.998	**1.333**	**1.015**	**0.996**	1.763	1.411	0.994	**1.416**	**1.100**	**0.996**

All features are from the thigh; thus, the “leg” in the “upper leg” (thigh) is omitted in intralimb synergy modeling for convenience. The *x*, *y*, and *z* in the subscript stand for the direction. For example, *ɷ_upx_* stands for the angular velocity of the thigh in *x* direction. The best result of each model is shown in bold.

**Fig. 5.**
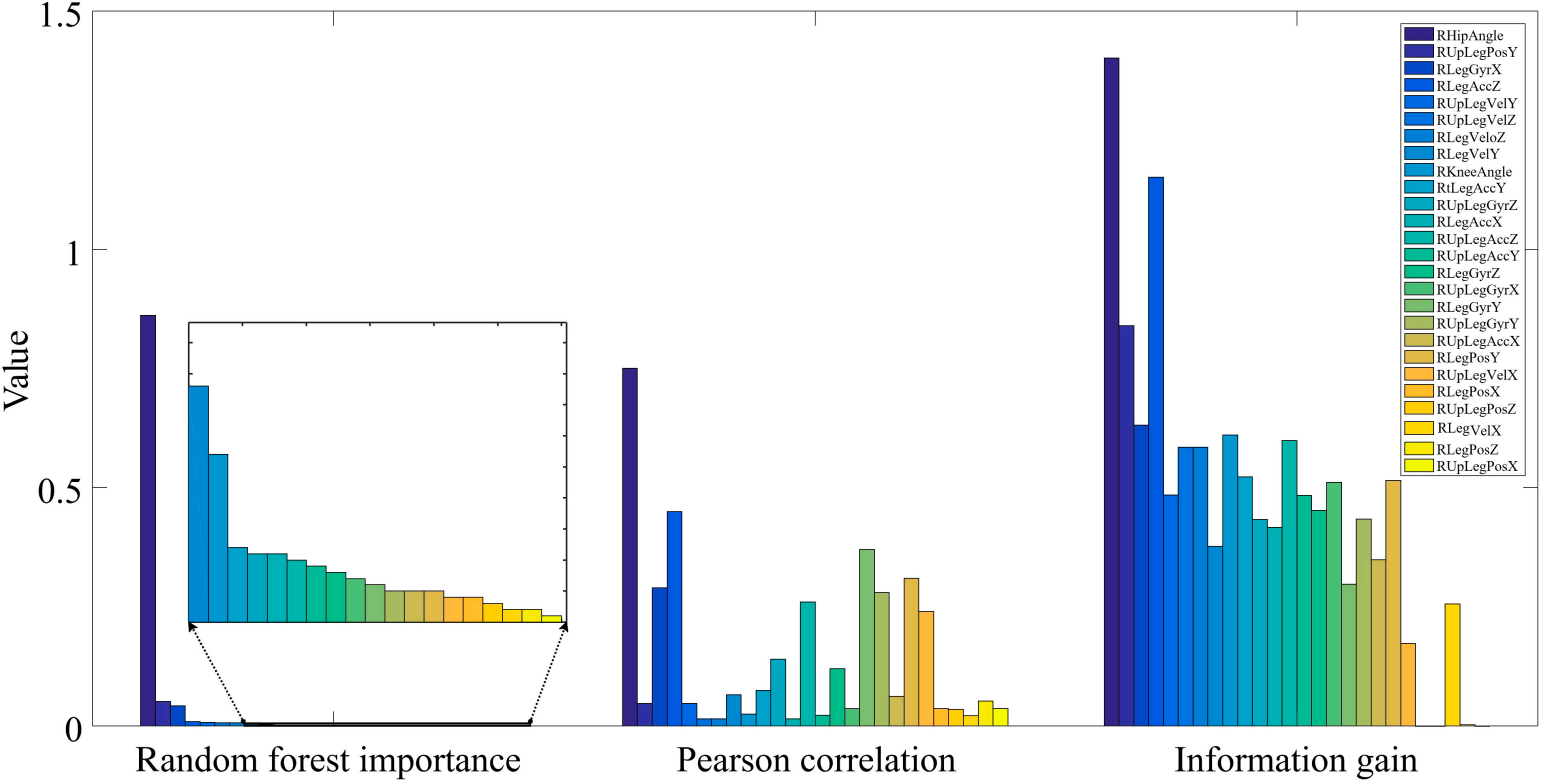
FS results of the 3 methods for interlimb synergy modeling. Four of the top 7 features selected by both the random forest and information gain methods are the same.

For interlimb synergy modeling, we need to select suitable inputs from a pool of 26 features [accelerations (*a*), velocities (*V*), positions (*x*), and angular velocities (*ω*) of the right shank and thigh in 3 directions of 2 limbs, with right hip and knee angles of the contralateral limb). Figure 5 presents the FS results using the random forest algorithm, Pearson correlation, and information gain. Only the top 7 features (threshold: random forest importance > 0.006, Pearson correlation > 0.25, and information gain > 0.5845) with the highest importance or correlations or information gain are displayed. It is worth noting that 4 of the top 7 features selected by both the random forest and information gain methods are the same. Similarly, to determine the optimal combinations of features, experiments based on the selected features (*n* = 1 to 7) suggested by the 3 methods were conducted (Table 4). A total of 18 sessions, including experiments with all 26 features, were performed to explore different combinations of the features for interlimb synergy modeling. The results indicate that the LSTM and RNN models perform the best with 6 features (*θ_Hip_θ_Knee_a_z_a_upz_ω_x_x_upy_*), while the Seq2Seq and GRU models perform the best with 7 features each (*θ_Hip_a_z_a_upz_ω_x_ω_y_ω_upy_x_y_* and *θ_Hip_a_z_ω_x_V_z_V_upy_V_upz_x_upy_*, respectively).

**Table 4.** Average experimental results of different combinations of the features selected for interlimb synergy modeling

	Seq2Seq			LSTM			RNN			GRU		
Features	RMSE	MAE	*R* ^2^	RMSE	MAE	*R* ^2^	RMSE	MAE	*R* ^2^	RMSE	MAE	*R* ^2^
*θ_Hip_*	1.692	1.320	0.979	1.096	0.894	0.993	1.243	0.996	0.992	1.142	0.930	0.993
*θ_Hip_x_upy_*	0.620	0.466	0.998	1.175	0.951	0.993	1.219	1.024	0.993	1.168	0.924	0.993
*θ_Hip_a_z_*	0.556	0.422	0.999	1.144	0.918	0.993	1.203	1.006	0.992	1.145	0.926	0.993
*θ_Hip_x_upy_ω_x_*	0.546	0.423	0.999	1.011	0.806	0.995	1.092	0.889	0.995	1.020	0.806	0.995
*θ_Hip_a_z_ω_y_*	0.599	0.473	0.998	1.109	0.897	0.993	1.258	1.006	0.992	1.156	0.933	0.993
*θ_Hip_a_z_x_upy_*	0.600	0.462	0.999	1.168	0.944	0.993	1.210	1.009	0.993	1.224	0.990	0.993
*θ_Hip_x_upy_ω_x_a_z_*	0.582	0.447	0.998	0.900	0.722	0.996	0.944	0.758	0.995	0.905	0.715	0.996
*θ_Hip_a_z_ω_y_x_y_*	0.550	0.417	0.998	1.041	0.850	0.994	1.183	0.967	0.992	1.055	0.865	0.994
*θ_Hip_x_upy_ω_x_a_z_V_upy_*	0.675	0.536	0.998	0.785	0.633	0.997	1.090	0.878	0.995	0.830	0.653	0.996
*θ_Hip_a_z_ω_y_x_y_ω_x_*	0.667	0.516	0.998	0.879	0.689	0.996	1.069	0.871	0.994	0.933	0.730	0.996
*θ_Hip_x_upy_ω_x_a_z_θ_Knee_*	0.831	0.671	0.997	0.687	0.566	0.998	0.935	0.758	0.997	0.693	0.552	0.998
*θ_Hip_x_upy_ω_x_a_z_V_upy_V_upz_*	0.595	0.423	0.998	0.831	0.661	0.997	1.013	0.796	0.995	0.851	0.689	0.996
*θ_Hip_a_z_ω_y_x_y_ω_x_ω_upy_*	0.613	0.453	0.998	0.874	0.678	0.996	1.082	0.876	0.995	0.987	0.781	0.996
*θ_Hip_x_upy_ω_x_a_z_θ_Knee_a_upz_*	0.589	0.465	0.998	**0.645**	**0.524**	**0.998**	**0.870**	**0.712**	**0.997**	0.721	0.576	0.998
*θ_Hip_x_upy_ω_x_a_z_V_upy_V_upz_V_z_*	0.564	0.410	0.999	0.683	0.539	0.998	0.935	0.798	0.998	**0.684**	**0.544**	**0.998**
*θ_Hip_a_z_ω_y_x_y_ω_x_ω_upy_a_upz_*	**0.540**	**0.404**	**0.999**	0.874	0.677	0.996	1.101	0.890	0.995	0.937	0.734	0.996
*θ_Hip_x_upy_ω_x_a_z_θ_Knee_a_upz_V_z_*	0.568	0.434	0.998	0.660	0.535	0.998	0.918	0.751	0.997	0.705	0.557	0.998
All	0.546	0.419	0.999	0.787	0.660	0.998	1.096	0.923	0.996	0.932	0.800	0.998

The best result of each model is shown in bold.

### FS methods

Among the 3 FS methods used, information gain was deemed unnecessary for the synergy modeling problem, while random forest and Pearson correlation were proved to be valuable. For intralimb synergy modeling, the Seq2Seq and RNN models exhibited the lowest error and best fitness values with the input *ω_upx_ω_upy_ω_upz_V_upy_x_upy_*. These were the top 5 features suggested by the random forest algorithm (Fig. 4). For interlimb synergy modeling, the best combination of features (*θ_Hip_a_z_a_upz_ω_x_ω_y_ω_upy_x_y_*) for the Seq2Seq model is the top 7 features suggested by Pearson correlation (Fig. 5), while the optimal combination of features (*θ_Hip_a_z_ω_x_V_z_V_upy_V_upz_x_upy_*) for the GRU model is the top 7 features selected by the random forest algorithm. Thus, these observations indicate that information gain may not contribute to FS in synergy modeling problems.

### FS-Seq2Seq yields the best results for synergy modeling

**Fig. 6.**
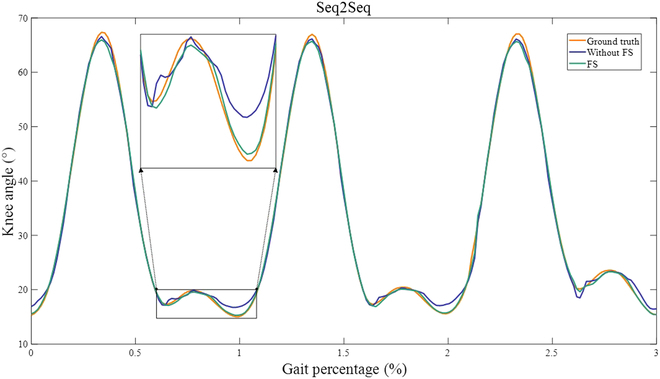
Partial experimental results of features with (green line) and without (blue line) FS in Seq2Seq’s intralimb synergy modeling.

**Fig. 7.**
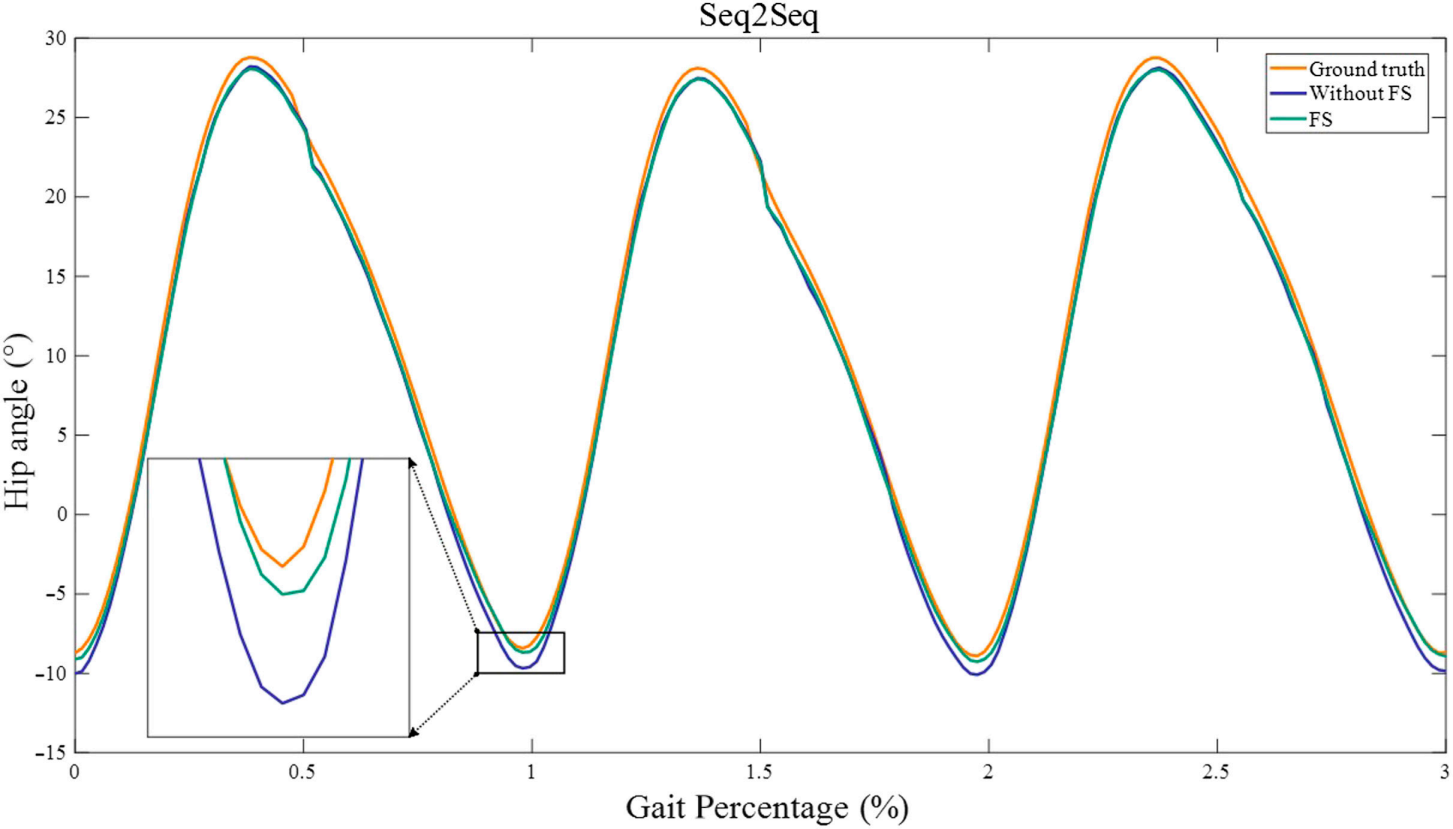
Partial experimental results of features with (green line) and without (blue line) FS in Seq2Seq’s interlimb synergy modeling.

The experimental results of the optimal combination of features for each of the modeling methods were compared with the results obtained using all features in the intralimb and interlimb synergy modeling experiments, respectively. Figures 6 and 7 demonstrate the improvements in the synergy modeling performance of the Seq2Seq model due to FS, and these improvements are significant according to a paired *t* test analysis (*P* < 0.05).

According to Table 5, Seq2Seq outperforms LSTM, RNN, and GRU in both interlimb and intralimb synergy modeling. Moreover, the proposed FS-Seq2Seq yields the best results. For intralimb synergy modeling, FS-Seq2Seq produced RMSE (0.859°), MAE (0.596°), and *R*^2^ (0.998) values: For interlimb synergy modeling, FS-Seq2Seq produced the RMSE (0.540°), MAE (0.404°), and *R*^2^ (0.999). In addition, we used a paired *t* test to compare the modeling errors obtained from FS-Seq2Seq with those of the other 3 models. The statistical analyses revealed significant differences between FS-Seq2Seq and the other 3 models. The results highlight the efficacy of the FS-Seq2Seq in both intralimb and interlimb synergy modeling tasks.

**Table 5.** Results of different methods for intralimb and interlimb synergy modeling (the FS-Seq2Seq yields the best result)

	Intralimb synergy modeling			Interlimb synergy modeling		
Methods	RMSE	MAE	*R* ^2^	RMSE	MAE	*R* ^2^
Seg2Seq	0.902	0.631	0.998	0.546	0.419	0.999
FS-Seq2Seq	0.859	0.596	0.998	0.540	0.404	0.999
LSTM	1.333	1.015	0.996	0.787	0.660	0.998
FS-LSTM	1.452	1.093	0.995	0.645	0.524	0.998
RNN	1.763	1.411	0.994	1.096	0.923	0.996
FS-RNN	1.520	1.189	0.994	0.870	0.712	0.997
GRU	1.416	1.100	0.996	0.932	0.800	0.998
FS-GRU	1.523	1.184	0.995	0.684	0.544	0.998

### Comparisons with existing studies

Table 6 summarizes the comparison between our results and those from prior studies (9 studies as also included in Table 2). In this study, FS-Seq2Seq yielded the highest performance for interlimb synergy modeling, achieving an RMSE of 0.540°, an MAE of 0.404°, and *R*^2^ of 0.999. These results surpass those reported for RNN [58], FFNN [27], and our previous work from 2018 [15]. Furthermore, the interlimb synergy LSTM model demonstrates improvement over our previous work [15]. This improvement can be attributed to the utilization of a larger sample size (*n* = 16) and additional FS for identifying the optimal combination of features as inputs. In 2021, Zou et al. [28] proposed a Seq2Seq interlimb model and reported an MAE of 0.50° after training for 500 epochs, reporting superior performance using LSTM. Here, we arrive at the same conclusion that Seq2Seq outperforms LSTM for interlimb synergy modeling. The experimental results of our FS-Seq2Seq model show lower MAE than that reported by Zou et al., which can be attributed to 3 reasons. First, it should be noted that FS was not performed in the study of Zou et al. [28]. Second, the use of 500 epochs as in the experiment of Zou et al. [28] could lead to overfitting issues and high computational demands. To address these issues, we chose to set the number of epochs to 10 in our experiment; otherwise, it is difficult to update the parameters online in the embedded computers. Last, the experiment of Zou et al. was based on data from only 3 subjects, whereas our database comprises a larger cohort. Therefore, we strongly advocate for FS-Seq2Seq, as a 2-stage approach to interlimb synergy modeling for generating trajectories in lower limb assistive devices. It is important to emphasize that FS plays a crucial role in mitigating model complexity and enhancing performance.

**Table 6.** Comparison of different synergy modeling methods from different studies

	Synergy	Methods	FS	RMSE(°)	MAE(°)	R^2^
Chereshnev 2018 [58]	Interlimb	RNN			4.99	
Liang 2018 [15]	Interlimb	LSTM		2.23		
Liu 2016 [60]	Interlimb and intralimb	LSTM			~1.5	
Lim 2019 [27]	Interlimb and intralimb	FFNN				0.90
Zou 2021 [28]	Interlimb	Seq2Seq			0.50	
**This work**	**Interlimb**	**Seq2Seq**	**Comprehensive**	**0.540**	**0.404**	**0.999**
Bennett 2013 [36]	Intralimb	ANN	Not thorough	3.90		0.97
Eslamy 2020 [37]	Intralimb	Gaussian process regression	Not thorough	6.30	5.10	0.89
Liang 2021 [1]	Intralimb	LSTM		3.89		0.94
Rai 2019 [59]	Interlimb and intralimb	LSTM		~7		
**This work**	**Intralimb**	**Seq2Seq**	**Comprehensive**	**0.859**	**0.596**	**0.998**

In terms of intralimb synergy modeling, our FS-Seq2Seq model yielded an RMSE of 0.859°, an MAE of 0.596°, and *R*^2^ of 0.998, while the optimal LSTM model achieved an RMSE of 1.333°, an MAE of 1.015°, and *R*^2^ of 0.996. These results further demonstrate Seq2Seq’s superior performance over LSTM. In addition, it should be noted that the results outperform those obtained using RNN, GRU, artificial neural network (ANN) [36], Gaussian process regression [37], LSTM by Rai et al. [59], and our previous work from 2021 [1]. It is important to acknowledge that FS was not comprehensively conducted in our previous work: consequently, FS-Seq2Seq is recommended for intralimb synergy modeling for trajectory generation in lower limb assistive devices.

## Conclusion

The concept of synergy holds great promise in trajectory prediction for assistive devices. On the one hand, trajectory prediction based on interlimb synergy has been proposed for rehabilitative exoskeletons to generate synergic reference trajectories for different patients with stroke to provide adaptive guidance, encourage participation, and help with rehabilitation. On the other hand, trajectory prediction based on intralimb synergy has been proposed for partial limb exoskeletons and above-knee prostheses to generate synergic reference trajectories in line with the wearer’s movements for autonomous control. However, no comprehensive comparisons have been conducted, and no optimal conclusions have been obtained from existing studies regarding the best synergy modeling method. Further, FS processes in the existing studies were often limited and incomplete.

The present study aimed to identify the optimal modeling method and feature combinations for modeling interlimb and intralimb synergies with the goal of generating desirable trajectories for control of lower limb assistive devices. Accordingly, a model was trained using gait data from 16 able-bodied subjects. The training process used the leave-one-out cross-validation approach, whereby gait data of one randomly selected subject were set as the test data and those of the remaining 15 subjects were used for training. The selected subject’s joint angles were estimated on the basis of the synergies modeled from the training data (remaining 15 subjects’ data). The results demonstrated that the Seq2Seq model outperformed GRU, RNN, and LSTM for both intralimb and interlimb synergy modeling. In terms of interlimb synergy modeling, Seq2Seq’s best model was better than RNN [58], FFNN [27], and LSTM models without FS [15] used in prior studies. We obtained the same conclusion as the study of Zou et al. [28] that Seq2Seq outperforms LSTM in interlimb synergy modeling. For intralimb synergy modeling, Seq2Seq showed better performance than ANN [36], Gaussian process regression [37], and LSTM without FS [1]. On the basis of these results, Seq2Seq models are recommended for both interlimb and intralimb synergy modeling. The utilization of Seq2Seq models offers advantages over other methods in terms of performance and accuracy. Further, considering the range of motion of the knee joints, the MAEs of 0.404° and 0.596° respectively obtained for the best interlimb and intralimb synergy model (FS-Seq2Seq) show the promise of applying synergy-based trajectory generation to assistive devices in practice.

Importantly, FS plays a crucial role in improving model performance. Note that there is no universal FS method that can be applied to all problems or datasets [44]. Different FS methods fit different specific problems. In this study, 3 state-of-the-art and commonly used methods (Pearson correlation, random forest, and information gain) were used for systematic FS for both interlimb and intralimb synergy modeling. Moreover, for our specific problem, identifying the appropriate number of features to terminate the FS procedure is challenging since the optimal stopping criteria are unknown. Thus, we performed experiments with different combinations of the selected features suggested by the 3 methods, including using all the features. Our findings suggest that random forest and Pearson correlation are valuable for FS in this study. First, in intralimb synergy modeling, Seq2Seq and RNN had the best fitness with the features *(ω_upx_ω_upy_ω_upz_V_upy_x_upy_*) selected by random forest. This means that the angular velocities in the 3 directions as well as positions and velocities in the *y* direction (upward) of the thigh contribute most to knee angle estimation based on intralimb synergy. Second, in interlimb synergy modeling, the best Seq2Seq model is achieved with the features (*θ_Hip_a_z_a_upz_ω_x_ω_y_ω_upy_x_y_*) suggested by Pearson correlation. However, we observed that information gain did not contribute to the FS process. Our results further emphasize the importance of conducting systematic FS before synergy modeling in future studies.

Overall, FS-Seq2Seq as a 2-stage strategy offers advantages over other approaches in terms of performance and accuracy for modeling interlimb and intralimb synergies. This study emphasizes the promise of synergy-based trajectory prediction for assistive devices to achieve synergic and user-adaptive trajectories that improve human–machine interactions. Future research efforts should hence continue to explore and refine these techniques to further improve assistive device control.

## Data Availability

The data included in this study are available upon reasonable request by contact with the corresponding author.
